# Pulmonary arterial hypertension associated with interferon therapy: a population-based study

**DOI:** 10.1186/s40248-016-0082-z

**Published:** 2017-01-17

**Authors:** Ravikanth Papani, Alexander G. Duarte, Yu-li Lin, Yong-Fang Kuo, Gulshan Sharma

**Affiliations:** 1 Division of Pulmonary, Critical Care and Sleep Medicine, University of Texas Medical Branch, John Sealy Annex 5.140, 301 University Boulevard, Galveston, TX 77555 USA; 2 Office of Biostatistics, Department of Preventive Medicine and Community Health, University of Texas Medical Branch, 301 University Boulevard, Galveston, TX USA

**Keywords:** Pulmonary arterial hypertension, Interferons, Drug toxicity

## Abstract

**Background:**

Isolated cases of pulmonary arterial hypertension (PAH) with interferon α or β therapy have been reported, but no population-based estimates of the incidence of the disease after interferon exposure are available. The aim of this study was to determine the incidence of PAH after initiation of interferon therapy, using a large commercial insurance database.

**Methods:**

Using National Drug Codes (NDCs) and Healthcare Common Procedure Coding System (HCPCS) codes, we utilized the Clinformatics™ Data Mart (CDM) database to identify subjects between 20 and 65 years old who received α or β interferon therapy between April 2001 and December 2012. Patients were followed from one year prior to the first medication claim for interferon to the first diagnosis of pulmonary hypertension using ICD-9-CM codes 416.0 and 416.8, or disenrollment. In those subjects diagnosed with pulmonary hypertension, a prescription for PAH-specific medications was used as a surrogate endpoint.

**Results:**

We identified 20,113 subjects who received interferon therapy during the study period. The median follow-up was 20 months. Pulmonary hypertension occurred in 71 subjects, and PAH-specific medications were prescribed to 7 of these subjects.

**Conclusion:**

Although our analysis showed that the development of PAH is a rare event with interferon therapy, the risk of developing the disease is several fold higher than that for the general population.

## Background

In the last quarter of the 20^th^ century, type I interferons, including α and β, were introduced as treatment for malignancies, chronic viral infections, and chronic neurologic conditions [1, 2]. Type I interferons are a family of glycoproteins with potent antiviral, immunomodulatory, and antitumor properties that are naturally generated in the human body, in response to pathogens and tumor cells. Consequently, interferon α was evaluated for the treatment of chronic hepatitis C with reports of sustained virologic response with prevention of further hepatic fibrosis and progressive organ failure [3–5]. In the management of relapsing multiple sclerosis, interferon β has been reported to decrease relapse rates and reduce disease burden when assessed by brain imaging [6, 7].

Although the benefits of interferon therapy are well documented, this medication has several side effects. It is commonly associated with flu-like illness and less often with neuropsychiatric effects (depression) and myelosuppression (pancytopenia). Occurrence of these side effects leads to dose reduction or delays in subsequent treatment and often resolve with drug discontinuation. Pulmonary arterial hypertension (PAH), a rare but serious side effect of interferon therapy, was first reported in a patient with renal cancer [8]. As the indications for interferon expanded to include chronic hepatitis and multiple sclerosis, the frequency of reported cases of PAH increased [9–19]. Hence, a consensus group of experts categorized interferon α and β as medications as possible risk factors for the development of PAH [20]. To investigate this association further, we developed a study with the aim of determining the incidence of PAH in a national population of patients living in the USA who were treated with α and β interferon therapy.

## Methods

### Study design and data source

This retrospective cohort study was conducted using the Clinformatics™ Data Mart (CDM) Database (OptumInsight, Eden Prairie, Minnesota, USA), which contains data on 53 million private insurance enrollees in the USA. The database contains medical claims, pharmacy claims, and administrative data (member file) for the enrollees. Because our study was a secondary data analysis, it was classified as exempt research by the University of Texas Medical Branch Institutional Review Board (IRB).

### Study cohort

**Fig. 1:**
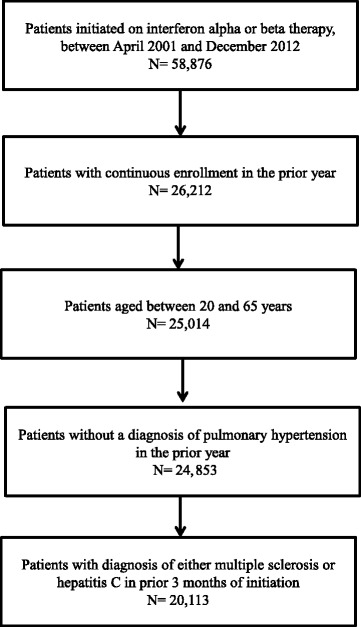
Cohort selection in patients treated with alpha or beta interferons

We initially identified subjects who were started on interferon α or β treatment between April 2001 and December 2012 (Fig. 1). From this cohort, we selected subjects with continuous enrollment in the prior year. We then selected those between 20 and 65 years old at treatment initiation. Finally, we identified subjects diagnosed with multiple sclerosis or hepatitis C in the 3 months before the start of interferon treatment. We excluded subjects with a prior diagnosis of pulmonary hypertension. We used International Classification of Diseases, Ninth Revision, Clinical Modification (ICD-9-CM) codes to identify patients with multiple sclerosis (340), hepatitis C (070.41, 070.44, 070.51, 070.54, 070.70, and 070.71), and pulmonary hypertension (416.0 and 416.8), respectively. Interferon treatment was identified using National Drug Codes (NDCs) and Healthcare Common Procedure Coding System (HCPCS) codes J1825, J1826, J1830, J9212, J9213, J9214, J9215, J9216, Q3025, Q3026, S0145, S0146, and S0148. NDCs for interferons were identified with use of the RED BOOK™ drug database (Truven Health Analytics Inc., Ann Arbor, Michigan, USA).

### Measures

Age, gender, region, and follow-up time were obtained from the member file. We used claims for medical services in the year prior to interferon initiation to determine comorbidities, including connective tissue disorders, liver disease other than hepatitis C, HIV/AIDS, obesity, hypertension, congestive heart failure, valvular heart disease, chronic pulmonary disease, diabetes, and sleep apnea (Appendix).

### Outcomes

The primary outcome of interest was PAH, defined as new initiation of PAH-specific therapies following interferon therapy. The occurrence of pulmonary hypertension was identified as the first ICD-9-CM code (416.0 or 416.8) registered following interferon therapy. Among those who developed the condition, we determined whether or not they received PAH-specific therapy, including ambrisentan, bosentan, epoprostenol, iloprost, macitentan, riociguat, sildenafil, tadalafil, and treprostinil. These medications were identified using NDCs and HCPCS codes J1325, Q4080, Q4074, J3285, Q4077, J7686, S0114, and S0090.

### Statistical analysis

We determined the number and proportion of study subjects with each of the aforementioned measures. We then estimated the percent of subjects developing pulmonary hypertension by the Kaplan-Meier method and presented the result in graphics. Among those with a diagnosis of pulmonary hypertension, we estimated the proportion of subjects receiving PAH-specific medications by the Kaplan-Meier method. Cox proportional hazards regression was used to examine the factors associated with pulmonary hypertension. All statistical analyses were performed using SAS version 9.3 (SAS Inc., Cary, North Carolina, USA). The significance level was set at 0.05.

## Results

We identified 20,113 subjects treated with interferon therapy for either hepatitis C or multiple sclerosis between April 2001 and December 2012 (Fig. 1). Approximately two-thirds of the patients treated with interferon α or β had hepatitis C (Table 1). Mean age was 46.4 ± 9.2 years, and the majority of the patients were between 40 and 59 years old. Gender distribution was similar. Common comorbid conditions included hypertension and diabetes observed in 18 and 8%, respectively. Other chronic liver conditions were recorded in 13% of the group and included concomitant hepatitis B and alcohol-related liver disease. One in five patients on interferon therapy had at least one of the following cardiovascular conditions: hypertension, congestive heart failure, valvular heart disease, and atrial fibrillation. Other conditions associated with pulmonary hypertension—such as chronic lung disease, obstructive sleep apnea, connective tissue disorders, and HIV/AIDS—were less common. The median follow-up time was 20 months (mean 29.49 ± 27.41).

**Table 1:** Baseline characteristics of patients newly initiated on interferon therapy between 2001 and 2012

Patient characteristics	Number	Percent
Indication for interferon		
Multiple sclerosis	7190	35.75
Hepatitis C	12,923	64.25
Age		
20–29	1205	5.99
30–39	3154	15.68
40–49	7526	37.42
50–59	7084	35.22
60–65	1144	5.69
Gender		
Male	9906	49.25
Female	10,207	50.75
Comorbidity^a^		
Hypertension	3612	17.96
Other liver disease^b^	2647	13.16
Diabetes	1589	7.90
Chronic pulmonary disease	750	3.73
Sleep apnea	338	1.68
Connective tissue disorder	331	1.65
Obesity	294	1.46
HIV/AIDS	268	1.33
Valvular heart disease	156	0.78
Congestive heart failure	86	0.43
Atrial fibrillation and flutter	74	0.37
Congenital heart disease	8	0.04
	Mean ± STD (Median)	
Age	46.36 ± 9.24 (48.00)	
Follow-up time, months	29.49 ± 27.41 (20.00)	

^a^A patient could have more than one comorbidity. ^b^Excluding hepatitis C

A total of 71 patients developed pulmonary hypertension during the study period, including 60 with hepatitis C and 11 with multiple sclerosis. The mean age for this group was 52.4 ± 33.5 years, with males comprising 57.7% of the cohort. Common comorbidities included chronic liver disease other than hepatitis C (42.3%), hypertension (33.8%), and diabetes mellitus (22.5%). The mean follow-up time for this group was 52.2 ± 33.5 months.

**Fig. 2:**
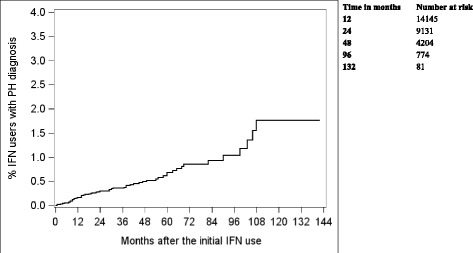
Time to the first diagnosis of pulmonary hypertension diagnosis after interferon treatment. The percentages of patients diagnosed with pulmonary hypertension by year 3, 6, and 9 were 0.36, 0.86 and 1.77%, respectively. IFN- interferons; PH- pulmonary hypertension

Figure 2 presents the time to first diagnosis of pulmonary hypertension in this cohort, estimated by the Kaplan-Meier method, after initiation of interferon therapy. At 3, 6, and 9 years of follow-up time, 0.36, 0.86, and 1.77% of patients developed pulmonary hypertension, respectively. Table 2 shows the factors associated with the development of pulmonary hypertension based on Cox proportional hazards regression. The odds ratio (OR) of developing pulmonary hypertension was 4.32 (95% CI 1.71–10.96) in patients with connective tissue disorders. Additional conditions associated with development of pulmonary hypertension included other liver disease excluding hepatitis C (OR 3.21; 95% CI 1.93–5.34), valvular heart disease (OR 3.76; 95% CI 1.16–12.21), and diabetes (OR 2.27; 95% CI 1.25–4.11).

**Table 2:** Factors associated with the likelihood of developing pulmonary hypertension

Patient characteristics	Hazard ratio (95% CI)
Indication for interferon	
Multiple sclerosis	Reference
Hepatitis C	1.43 (0.69, 2.96)
Age	
20–29	Reference
30–39	0.26 (0.02, 4.23)
40–49	1.46 (0.19, 11.19)
50–59	2.41 (0.32, 18.29)
60–65	4.05 (0.49, 33.36)
Gender	
Male	Reference
Female	1.02 (0.62, 1.68)
Comorbidity, Yes vs No	
Hypertension	1.43 (0.84, 2.43)
Other liver disease^a^	3.21 (1.93, 5.34)
Diabetes	2.27 (1.25, 4.11)
Chronic pulmonary disease	1.92 (0.82, 4.50)
Sleep apnea	1.31 (0.31, 5.56)
Connective tissue disorder	4.32 (1.71, 10.96)
Obesity	0.73 (0.10, 5.51)
HIV/AIDS	2.63 (0.63, 10.89)
Valvular disease	3.76 (1.16, 12.21)
Congestive heart failure	1.10 (0.15, 8.17)

Note: Congenital heart disease and atrial fibrillation and flutter were not included in the model because there were no events among patients with such comorbidities. This model was adjusted for the year of the initial interferon treatment. ^a^Does not include hepatitis C

**Fig. 3:**
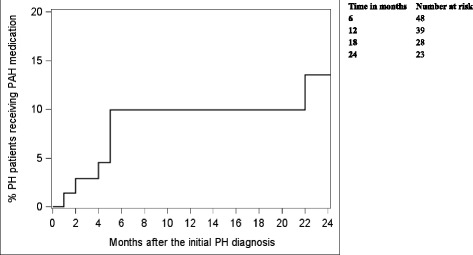
Time to the first PAH medication after the first diagnosis of pulmonary hypertension. By the end of follow-up, 7 patients received medication, 6 out of them did so within the fifth month. IFN-interferons; PH-pulmonary hypertension

Among the 71 patients who developed pulmonary hypertension, 7 were treated with PAH-specific therapies, and 6 out of them were treated within five months from their diagnosis (Fig. 3). Treatment medications included sildenafil (*n* = 4), tadalafil (*n* = 1), epoprostenol (*n* = 1), and inhaled iloprost (*n* = 1).

## Discussion

In this retrospective, administrative claims study of US patients with hepatitis C and multiple sclerosis treated with interferon, a new diagnosis of pulmonary hypertension was recognized in 71 individuals, and 7 patients were prescribed PAH-specific therapies. At first glance, the incidence appears low. However, when compared to the baseline risk in a general population, the incidence is quite substantial. To elaborate, the total person-years in our study cohort of 20,113 subjects was 49,360.5 person-years based on the follow-up time. Considering that the estimated incidence of PAH is 1.1 to 7.6 per million adults per year [21], we should have observed less than one PAH case in our study cohort. However, we identified 7 cases. This suggests that the risk of developing PAH in patients receiving interferon α or β therapy is several fold higher than in general population.

Several drugs and toxins have been recognized as risk factors in the development of PAH and have been categorized based on the strength of evidence as definite, likely, possible, and unlikely [20]. Epidemiologic studies have determined a definite association between appetite suppressants as well as contaminated rapeseed oil with the development of PAH [22–24]. A consensus group of experts reviewed the evidence regarding the risk associated with development of PAH and designated α and β interferons as possible risk factors [20]. In support of this, case reports have described new-onset PAH with interferon administration for treatment of chronic myelogenous leukemia, renal cell cancer, melanoma, chronic hepatitis, and multiple sclerosis [8–19]. Savale and colleagues described patients with newly diagnosed or worsening PAH associated with interferon therapy, from the French PAH registry [25]. In their case series, the majority of patients received interferon for treatment of chronic hepatitis C and hemodynamic measurements were performed to confirm the diagnosis. In addition, the majority of patients carried other risk factors for PAH, namely portal hypertension and/or HIV infection. Similarly, our study represents patients with hepatitis C and multiple sclerosis treated with interferon that developed pulmonary hypertension and subsequently received PAH-specific therapies. These reports support the association of α and β interferons in the development of PAH.

There is a growing body of evidence implicating the role of inflammation and autoimmunity in the development of PAH, and this has produced work examining the role of interferons in pulmonary vascular pathology [26, 27]. Endothelin-1 is a well-established mediator in the pathogenesis of PAH and is overexpressed in patients with PAH [28]. Elevated endothelin was found in patients receiving interferon α therapy for chronic hepatitis C, and the dependent increase in serum endothelin levels seen in these patients was related to interferon and not to the virus [29]. A group of investigators systematically addressed the role of interferon in PAH, using in vitro and in vivo experimental models as well as clinical samples from patients with scleroderma with and without PAH [30]. They noted that type I interferons induced endothelin-1 release from human pulmonary artery smooth muscle cells. Mice lacking functional type I interferon receptor (IFNAR1^−/−^) were protected from the effects of hypoxia and development of PAH. When clinical samples were analyzed, a greater number of scleroderma patients with PAH had detectable levels of interferons, along with significantly higher levels of endothelin-1, when compared to patients without PAH. Apart from elevated serum levels of interferons and endothelins, interferon receptor expression was also increased in lung sections of scleroderma patients with PAH. Thus, investigators concluded that type I interferons, their receptors, and downstream mediators are associated with PAH. Collectively, these reports provide an underlying mechanism by which type I interferon results in the development of PAH.

Our study has several limitations. An important limitation concerns the use of ICD-9 codes for the diagnosis of PAH. Prior investigations have indicated that these codes do not adequately distinguish PAH from non-PAH patients [31–33]. To address this issue, we only examined patients who were treated with PAH-specific therapies, thereby focusing on patients for whom the treating physician had sufficient evidence to prescribe PAH-specific therapies. In addition, we identified patients with continuous enrollment lacking an ICD-9 code diagnosis for pulmonary hypertension in the preceding one year. While, hemodynamic data were not available in this database, we believe that a prescription for PAH-specific therapy indicates a provider’s clinical diagnosis of PAH. We assume that an ICD code and subsequent prescription for PAH-specific therapy represents a “real world” diagnosis of PAH. Another limitation concerns the duration of follow-up. Our data was extracted from a large insurance claims database, and enrollees often discontinued their insurance policies when they switched to different employers who purchase insurance plans from other insurance carriers. Therefore, the dropout rate was high. Reports regarding interferon-induced PAH indicate that onset of the pulmonary vascular disease process may take up to 5 years, and our study may underestimate the incidence. Lastly, although our study cohort had several comorbidities that are independent risk factors for PAH, their prevalence in the cohort was much less than in general population.

## Conclusion

Using an administrative claims database, we found that the frequency of PAH in interferon-treated hepatitis C or multiple sclerosis patients was several fold higher than that for the general US population.

## Appendix

**Table 3:** ICD-9-CM codes used for identifying comorbidities

Comorbidity	ICD-9-CM
Connective tissue disorder	446, 701.0, 710.0, 710.1, 710.2, 710.3, 710.4, 710.8, 710.9, 711.2, 714, 719.3, 720, 725, 728.5, 728.89, 729.30
Other liver disease	070.22, 070.23, 070.32, 070.33, 070.6, 070.9, 456.0, 456.1, 456.2, 570, 571, 572.2, 572.3, 572.4, 572.8, 573.3, 573.4, 573.8, 573.9, V42.7
HIV/AIDS	042, 043, 044
Obesity	278.0
Hypertension	401, 402, 403, 404, 405
Congestive heart failure	398.91, 402.01, 402.11, 402.91, 404.01, 404.03, 404.11, 404.13, 404.91, 404.93, 425.4, 425.5, 425.7, 425.8, 425.9, 428
Valvular disease	093.2, 394, 395, 396, 397, 424, 746.3, 746.4, 746.5, 746.6, V42.2, V43.3
Chronic pulmonary disease	416.9, 490, 491, 492, 493, 494, 495, 496, 500, 501, 502, 503, 504, 505, 506.4, 508.1, 508.8
Diabetes	250.0, 250.1, 250.2, 250.3, 250.4, 250.5, 250.6, 250.7, 250.8, 250.9
Congenital heart disease	746.9, 745.4
Atrial fibrillation and flutter	427.3
Sleep apnea	327.21, 327.23, 327.27, 780.51, 780.53, 780.57, 786.03, 786.04

For each of the comorbidities, the study subjects needed to have 2 claims separated by at least 30 days in the year before the interferon initiation to be considered to have that comorbidity
